# Bacterial extracellular vesicles affect endocrine therapy in MCF7 cells

**DOI:** 10.1097/MD.0000000000025835

**Published:** 2021-05-07

**Authors:** Jeongshin An, Jong Bin Kim, Eun Yeol Yang, Hye Ok Kim, Won-Hee Lee, Jinho Yang, Hyungju Kwon, Nam Sun Paik, Woosung Lim, Yoon-Keun Kim, Byung-In Moon

**Affiliations:** a Department of Surgery, Ewha Womans University School of Medicine, 1071 Anyangcheon-ro, Yangcheon-gu; b Research Center for Cellular Homeostasis, Ewha Womans University, 52 Ewhayeodae-gil, Seodaemun-gu; c Department of Nuclear Medicine, Ewha Womans University School of Medicine, 1071 Anyangcheon-ro, Yangcheon-gu; d MD Healthcare, Room 1303, Woori Technology Inc. building, Sangam-dong, World Cup Buk-ro 56-gil, Mapo-gu, Seoul, Republic of Korea.

**Keywords:** breast neoplasms, extracellular vesicles, *Klebsiella pneumoniae*, tamoxifen

## Abstract

**Background:**

: The microbiome is important in the development and progression of breast cancer. This study investigated the effects of microbiome derived from *Klebsiella* on endocrine therapy of breast cancer using MCF7 cells. The bacterial extracellular vesicles (EVs) that affect endocrine therapy were established through experiments focused on tamoxifen efficacy.

**Methods:**

: The microbiomes of breast cancer patients and healthy controls were analyzed using next-generation sequencing. Among microbiome, *Klebsiella* was selected as the experimental material for the effect on endocrine therapy in MCF7 cells. MCF7 cells were incubated with tamoxifen in the absence/presence of bacterial EVs derived from *Klebsiella pneumoniae* and analyzed by quantitative real-time polymerase chain reaction and Western blot.

**Results:**

: Microbiome derived from *Klebsiella* is abundant in breast cancer patients especially luminal A subtype compared to healthy controls. The addition of EVs derived from *K pneumoniae* enhances the anti-hormonal effects of tamoxifen in MCF7 cells. The increased efficacy of tamoxifen is mediated via Cyclin E2 and *p*-ERK.

**Conclusion:**

: Based on experiments, the EVs derived from *K pneumoniae* are important in hormone therapy on MCF7 cells. This result provides new insight into breast cancer mechanisms and hormone therapy using *Klebsiella* found in the microbiome.

## 1 Introduction

The incidence of breast cancer in Korea is rapidly increasing. In 1988, only 7.2% of all female cancers in Korea were diagnosed as breast cancer; in 2018, 23% of newly diagnosed cancers in women were breast cancer.^[1,2]^ Many studies have suggested that the increasing incidence of breast cancer is due to the westernization of dietary habits in Korea.^[3]^ These changes in eating habits have led to differences in the intestinal microbiome.^[4]^

Although there were differences according to the types of samples tested, there were clear differences between the microbiomes of breast cancer patients and healthy controls.^[5]^ The tissues of breast cancer patients have been shown to be abundant in *Fusobacterium*, *Atopobium*, *Gluconacetobacter*, *Hydrogenophaga*, and *Lactobacillus*.^[6]^ Nipple aspiration fluid was compared between breast cancer patients and healthy controls, and an unclassified genus from the *Sphingomonadaceae* family was found to be more common in healthy controls.^[7]^

Diverse microbiomes produce diverse metabolites, and these metabolites have been linked to the health and disease of the host. A total of 50% of serum metabolites originate from symbiotic bacteria in the human body.^[8]^ Therefore, understanding differences in the microbiome is needed in the development and treatment of breast cancer via bacterial compounds such as metabolites.

Various microbiomes have been shown to be important in cancer progression as well as in treatment. Many experiments have assessed the chemotherapeutic effects of antibiotics in germ-free mice.^[9]^ Certain intestinal bacteria such as *Enterococcus hirae* and *Barnesiella intestinihominis* showed longer progression-free survival in advanced lung and ovarian cancer patients treated with chemoimmunotherapy via immune modulatory effect.^[10]^ Treatment of mice with low dose amoxapine with CPT-11 reduced tumor growth, suggesting that certain bacteria are involved in cancer progression.^[11]^ However, to date, there are no studies on microbes involved in breast cancer and endocrine therapy.

This study investigated bacterial extracellular vesicles (EVs) that affect endocrine therapy in breast cancer based on South Korean microbiome data. *Klebsiella* is abundant in the luminal A subtype of breast cancer patients in South Korea; thus, *Klebsiella* may affect the endocrine therapy of breast cancer. The effect of bacterial EVs has been experimented in MCF7 cells with tamoxifen.

## 2 Materials and methods

### 2.1 Bacterial EV isolation and DNA extraction

Female urine samples were collected from 220 healthy individuals and 127 patients with breast cancer in Ewha Womans University Mokdong Hospital and Inje University Haeundae Hospital (Table 1). This study was approved by the Institutional Review Board at Ewha Womans University Hospital (IRB No. EUMC 2014-10-005-019) and Inje University Haeundae Hospital (IRB No. 1297992-2015-064). To separate EVs from urine, the urine samples were separated by centrifugation at 10,000 × *g* for 10 min at 4°C. After centrifugation, the supernatant was sterilized with a 0.22 μm filter to remove bacteria and foreign materials completely. To extract DNA from the EV membrane, the EVs were separated from the urine by boiling at 100°C for 40 min. The supernatant was centrifuged at 13,000 rpm at 4°C for 30 min to remove suspended particles and waste. EV DNA was extracted using a DNA isolation kit (PowerSoil DNA Isolation Kit, MO BIO Laboratories, Inc., Carlsbad, CA). The DNA from the EVs of each sample was quantified using the QIAxpert system (Qiagen, Hilden, Germany).

**Table 1:**
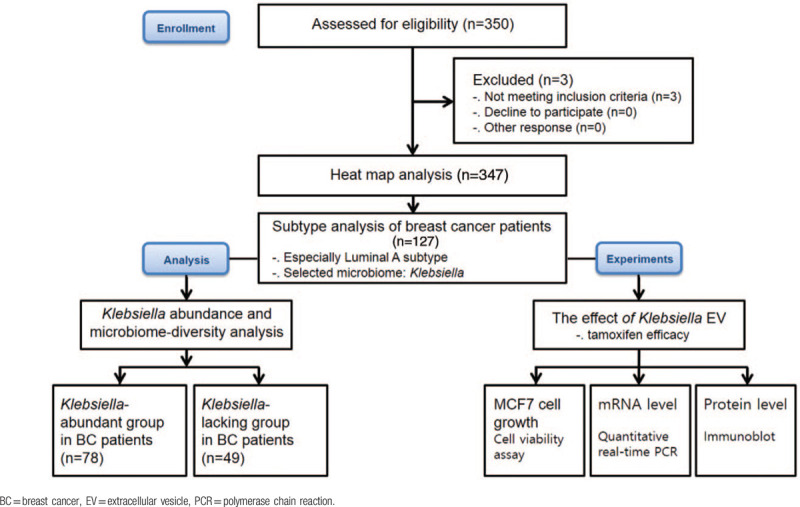
A study design flowchart: enrolment, analysis, and experiments of breast cancer patients.

### 2.2 Human microbiome analysis of urine samples

Metagenomic analysis was performed using next-generation sequencing. Bacterial genomic DNA was amplified using the V3-V4 hypervariable regions of 16S ribosomal DNA (rDNA) as primers: 16S_V3_F (5′-TCGTCGGCAGCGTCAGATGTGTATAAGAGACAGCCTACGGGNGGCWGCAG-3′) and 16S_V4_R (5′-GTCTCGTGGGCTCGGAGATGTGTATAAGAGACAGGACTACHVGGGTATCTAATCC-3′). According to the MiSeq System guide (Illumina, San Diego, CA), the libraries were prepared and QIAxpert (Qiagen) was used for quantification. According to the manufacturer's recommendations, each amplicon was quantified, set in an equimolar ratio, pooled, and sequenced with a MiSeq (Illumina).

### 2.3 Analysis of the bacterial composition of the microbiome

Raw pyrosequencing reads obtained from the sequencer were filtered according to the barcode and primer sequences using MiSeq (Illumina). Taxonomic assignment was performed using the profiling program MDx-Pro ver. 1 (MD Healthcare Inc., Seoul, Korea). The high-quality sequencing reads were selected after verifying the read length (300 bp) and quality score (average Phred score ≥20). Operational taxonomic units were clustered using the sequence clustering algorithm CD-HIT. Subsequently, the taxonomic assignment was carried out using UCLUST and QIIME against the 16S rDNA sequence database in GreenGenes 8.15.13. Based on the sequence similarities, all 16S rDNA sequences were assigned to the following taxonomic levels. The bacterial composition at each level was plotted in the stacked bar. In cases where clusters could not be assigned at the genus level due to lack of sequences or redundant sequences in the database, the taxon was assigned at a higher level, which is indicated in parentheses.

### 2.4 Extraction of *Klebsiella pneumoniae* EVs

**Figure 1:**
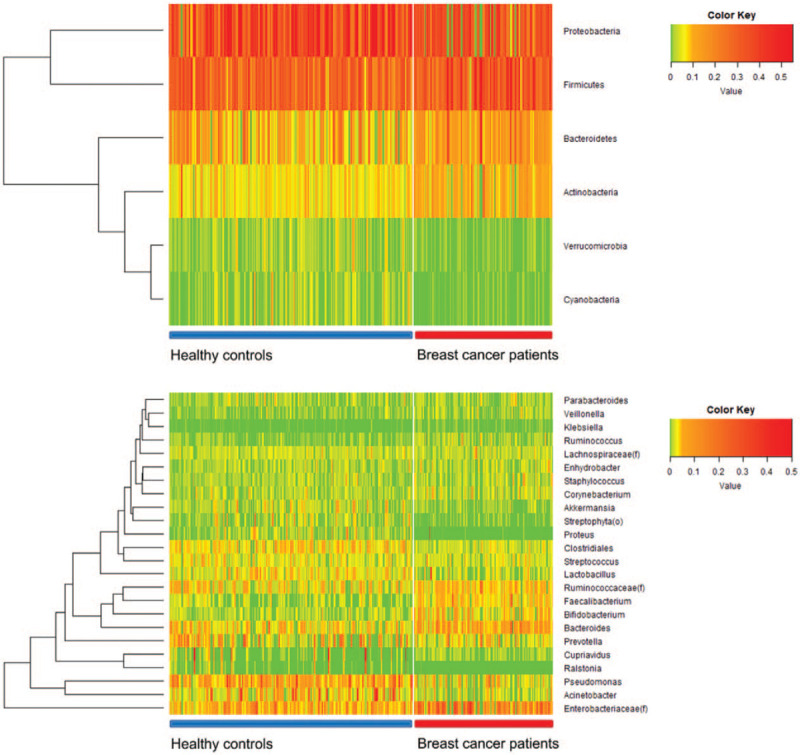
Heat map shows the relative abundance of different phyla and genera of the microbiomes of breast cancer patients and healthy controls. The upper heat map shows the difference in phyla and the lower heat map shows the difference in genera.

EVs were isolated from *K pneumoniae* as previously described.^[12]^ Bacteria were incubated in Luria–Bertani broth at 37°C with shaking at 200 rpm. A bottle top vacuum filter with a pore size of 0.45 μm (Corning, Inc., Corning, NY) was used to obtain the supernatant, which was then filtered using the QuixStand Benchtop System (Amersham Biosciences, Little Chalfont, UK). A bottle top vacuum filter with a pore size of 0.22 μm (Corning, Inc.) was used to remove any residual bacteria from the supernatant. EVs from *K pneumoniae* were harvested and confirmed by transmission electron microscopy (TEM). Images of the EVs were taken via a Hitachi (H7650, 80 kV) TEM (Fig. 1).

### 2.5 Cell viability assay

A total of 5 × 10^5^ MCF7 cells were seeded in Dulbecco modified Eagle medium supplemented with 10% fetal bovine serum in 4-well polystyrene culture plates (Nunc/Thermo Scientific, Rochester, NY). Following 24 hours of culture, cells were washed twice with phosphate-buffered saline (PBS), and fresh medium was added. The cells were treated with distilled water (control [CTL]) or EVs at 10, 100, or 1 μg/ml for 72 hours. EVs were diluted in distilled water to achieve the desired concentration. *K pneumoniae* EVs at 100 ng/ml were administered with tamoxifen, according to the titrated dose of *K pneumoniae*. The cells were treated with dimethyl sulfoxide (DMSO) (CTL) (Sigma-Aldrich, St. Louis, MO) and 10 μM tamoxifen, 100 ng/ml *K pneumoniae* EVs, or 10 μM tamoxifen and 100 ng/ml *K pneumoniae* EVs for 72 hours. The concentration of tamoxifen was chosen according to the results of previous studies. Viable cells were counted in a Neubauer chamber using a trypan blue viability assay. Relative cell survival is shown as the percentage of viable control cells.

### 2.6 Quantitative real-time PCR for signaling molecule analysis

MCF7 cells were harvested using trypsin-EDTA and washed with phosphate-buffered saline. Total RNA was extracted with the RiboEx [GeneAll, Seoul, Korea]. After this process, cDNA was synthesized using SuperScript III reverse transcriptase (Invitrogen, Carlsbad, CA). Quantitative real-time polymerase chain reaction (PCR) was performed using a QuantiTect SYBR Green PCR Kit (Qiagen, Valencia, CA). Table 2 shows primer sequences of signaling molecules.

**Table 2:** Primer sequences for qRT-PCR analysis of gene expression.

Gene	Type	Sequence	MER	TM	GC (%)	Size (bp)
CCND1	qRT-F	CTCTGTGCCACAGATGTGAAG	21	57.9°C	52	170
	qRT-R	GAGGCAGTCCGGGTCACAC	19	57.2°C	68	
CCND2	qRT-F	TGTGTGCCACCGACTTTAAG	20	55.0°C	50	172
	qRT-R	TTGAGACAATCCACGTCTGTG	21	55.3°C	48	
CCNE1	qRT-F	AGGTTTCAGGGTATCAGTGGTG	22	55.8°C	50	175
	qRT-R	GCTTTGTCCAGCAAATCCAAG	21	55.8°C	48	
CCNE2	qRT-F	CTGGCTTTTAGAGGTATGTGAAG	23	56.0°C	43	162
	qRT-R	AGCATAGATTTCCTCAAGTTTGG	23	55.3°C	39	
CCNA1	qRT-F	CTAAGTACGTAGCAGAGCTG	20	55.5°C	50	127
	qRT-R	GTTTCTGGCCAAAAGTGCTTG	21	55.7°C	48	
CCNA2	qRT-F	GGACAAAGCTGGCCTGAATC	20	55.9°C	55	166
	qRT-R	GGAGAGAAACACCATGATACTTTG	24	55.2°C	42	
CCNB1	qRT-F	ATAATGGTGAATGGACACCAACTC	24	55.1°C	42	143
	qRT-R	ATACTTGTTCTTGACAGTCATGTG	24	54.8°C	38	
CCNB2	qRT-F	AGCTGGAGGTTTTGCAGTC	19	54.8°C	53	184
	qRT-R	CGGGAAACTGGCTGAACCTG	20	57.9°C	60	
P21	qRT-F	ACCATGTGGACCTGTCACTG	20	54.8°C	55	135
	qRT-R	TGGAGTGGTAGAAATCTGTCATG	23	55.5°C	43	
P27	qRT-F	GACCTGCAACCGACGATTC	19	55.2°C	58	156
	qRT-R	TATTCTTAATTCGAGCTGTTTACG	24	55.3°C	33	
TNF	qRT-F	AGGCAGTCAGATCATCTTCTC	21	55.6°C	48	162
	qRT-R	CTGATGGCACCACCAGCTG	19	57.9°C	63	
GAPDH	qRT-F	GAGTCAACGGATTTGGTCG	19	57.5°C	53	133
	qRT-R	TGGAATCATATTGGAACATGTAAAC	25	57.8°C	32	

CCNA1 = Cyclin A1, CCNA2 = Cyclin A2, CCNB1 = Cyclin B1, CCNB2 = Cyclin B2, CCND1 = Cyclin D1, CCND2 = Cyclin D2, CCNE1 = Cyclin E1, CCNE2 = Cyclin E2, GAPDH = glyceraldehyde 3-phosphate dehydrogenase, P21 = cyclin-dependent kinase inhibitor 1, P27 = cyclin-dependent kinase inhibitor 1B, qRT-F = forward primer, qRT-PCR = quantitative real-time polymerase chain reaction, qRT-R = reverse primer, TM = temperature, TNF = tumor necrosis factor.

### 2.7 Immunoblotting

MCF7 cells were treated with DMSO (CTL) and 10 μM tamoxifen, 100 ng/ml *K pneumoniae* EVs, or 10 μM tamoxifen and 100 ng/ml *K pneumoniae* EVs for 72 hours. In accordance with the manufacturer's instructions (Cell Signaling Technology, Beverly, MA), total cell lysates were prepared in 200 μl of lysis buffer. A Bradford assay with the Bio-Rad Protein Assay kit (Bio-Rad Laboratories, Hercules, CA) was used to measure protein concentrations. Equal amounts of protein were separated by 10% SDS-PAGE (Bio-Rad Laboratories) and electrotransferred onto Hybond-ECL nitrocellulose membranes (Amersham Bioscience). Protein blots were immunolabeled with anti-phospho-AKT1/2/3 rabbit polyclonal antibody (1:1000, Santa Cruz Biotechnology), anti-phospho-ERK mouse monoclonal antibody (1:1000, Santa Cruz Biotechnology), anti-P21 mouse monoclonal antibody (1:1000, Santa Cruz Biotechnology), and anti-β-actin mouse monoclonal antibody (1:1000, Cell Signaling Technology). The blots were then washed with Tris-buffered saline with 0.2% Tween-20 (Sigma-Aldrich), and incubated for 1 hour at room temperature with peroxidase-conjugated AffiniPure rabbit anti-mouse IgG antibody (1:2500; Jackson ImmunoResearch Laboratories, West Grove, PA) or peroxidase-conjugated AffiniPure mouse anti-rabbit IgG (1:2500, Jackson ImmunoResearch Laboratories). Labeled proteins were detected using an enhanced chemiluminescence detection system (Amersham Biosciences).

### 2.8 Statistical analysis

The statistical significance of differences between groups was assessed using the Student *t*-test. *P* values of less than 0.05 were considered statistically significant. All of the experiments were performed three times including cell viability, quantitative real-time PCR, and immunoblotting. These data were statistically processed.

## 3 Results

### 3.1 Microbiomes of breast cancer patients were significantly different compared to healthy controls

Microbiome analysis of the EVs of breast cancer patients and healthy controls showed a significant difference between the two groups. *Bacteroides* and *Ruminococcaceae* were more common in the breast cancer group, and *Clostridiales* and *Pseudomonas* were relatively abundant in healthy controls. The EVs of the top 10 symbiotic bacteria, rich in breast cancer patients and healthy controls, were different (Fig. 1). In addition, subtype analysis was performed to identify symbiotic bacterial EVs, which are thought to affect breast cancer hormone receptors.

### 3.2 *Klebsiella* is abundant in the microbiome of the luminal A subtype of breast cancer

**Figure 2:**
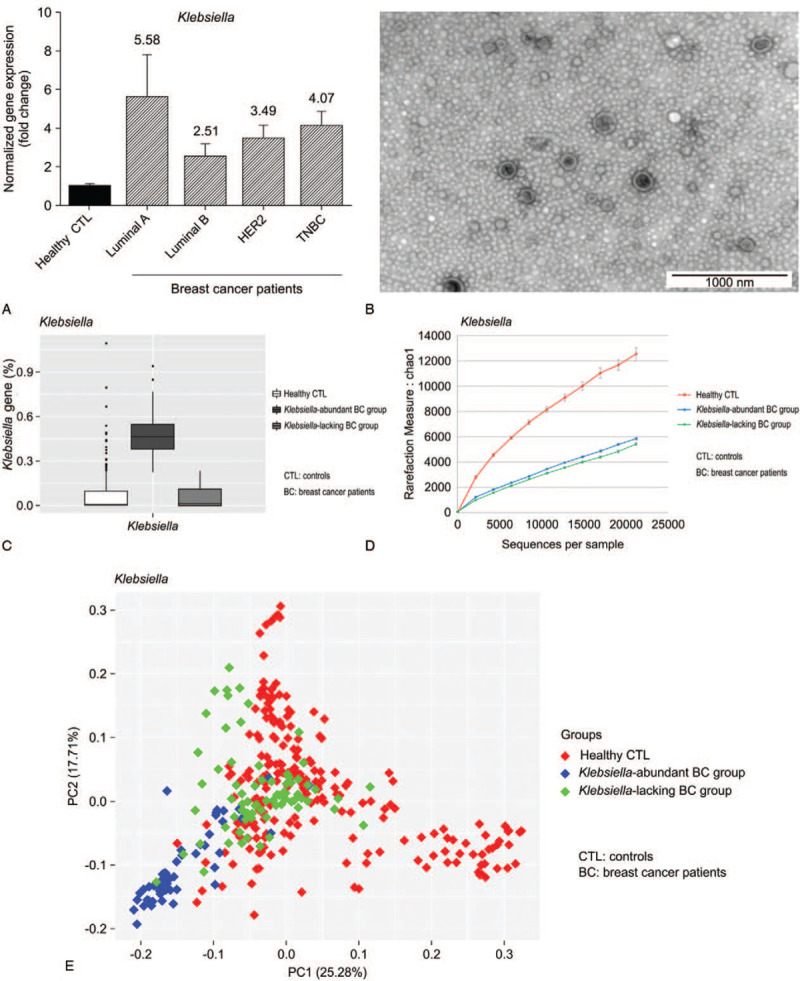
Relationship between hormone-positive breast cancer and *Klebsiella*. (A) *Klebsiella* is abundant in the microbiomes of luminal A subtype breast cancer patients. *Klebsiella* was 5.58-fold more abundant in breast cancer patients, especially in luminal A subtype patients. (B) *Klebsiella pneumonia* EVs were extracted by mass culture and confirmed by transmission electron microscopy. The average diameter of a *K pneumoniae* EV was approximately 30 nm. (C) Frequency of *Klebsiella* microbiome in healthy control and patient groups. Patients were divided into 2 groups according to the frequency of the *Klebsiella* microbiome. (D) Comparison of alpha diversity between the healthy control group and patient group. (E) Comparison of beta diversity between the healthy control group and patient group. EVs = extracellular vesicles.

Among breast cancer subtypes, the luminal A subtype is positive for estrogen and progesterone receptors and does not exhibit HER2 gene overexpression. *Klebsiella* EVs were abundant in the luminal A subtype of breast cancer. The abundance of *Klebsiella* was 5.58-fold higher in the luminal A subtype breast cancer group than in the healthy control group (Fig. 2A). *K pneumoniae* was selected among *Klebsiella* genera as a source of bacterial EVs. *K pneumoniae* was cultured and bacterial EV vesicles were extracted and confirmed by TEM (Fig. 2B). The breast cancer patients were separated based on the abundance of *Klebsiella*. The average *Klebsiella* EV amount in breast cancer patients was the standard score. The boxplot is shown according to *Klebsiella* EV amount in breast cancer patients and healthy controls (Fig. 2C). The α-diversity (Fig. 2D) and β-diversity (Fig. 2E) are shown by Chao1, PC1, and PC2. The breast cancer patients showed reduced microbiome diversity compared to the healthy controls. Based on the average value of the *Klebsiella* microbiome of breast cancer patients, the group of breast cancer patients with higher values than the average and those with lower values of breast cancer were divided (Fig. 2C to E).

### 3.3 *K pneumoniae* EVs did not affect the growth of estrogen receptor-positive breast cancer cells

**Figure 3:**
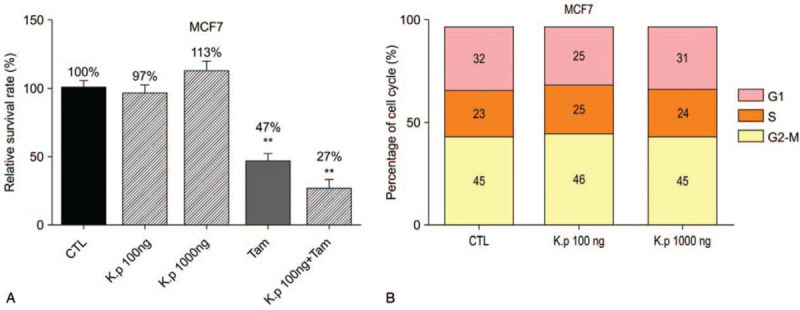
Treatment of MCF7 cells with *K pneumoniae* EVs or tamoxifen. Relative cell survival percentages following treatment with *K pneumoniae* EVs. The cells were treated with distilled water (CTL) or *K pneumoniae* EVs at 100 ng/ml or 1 μg/ml, DMSO (CTL) with 10 μM Tam, 10 μM Tam plus 100 ng/ml *K pneumoniae* EVs for 72 h. Surviving cells were counted in a Neubauer chamber. The percentages of viable cells are indicated over each bar. Relative cell survival percentages are shown as percentages of untreated viable cells. ∗∗*P* < 0.01. CTL = control, EVs = extracellular vesicles, Tam = tamoxifen.

To observe the effects of *K pneumoniae* EVs on the growth of estrogen receptor-positive breast cancer cells, the survival rate of MCF7 cells was determined following treatment with *K pneumoniae* EVs. Dose titration of *K pneumoniae* was performed; the final doses were 100 and 1000 μg/ml. The range of appropriate concentration was determined by measuring cell survival. The cell survival rate was different depending on the dose, but this difference was not statistically significant (Fig. 3). These results showed that *K pneumoniae* EVs do not inhibit the growth of estrogen receptor-positive breast cancer cells. *K pneumoniae* EVs also did not change the morphology of MCF7 cells.

### 3.4 *K pneumoniae* EVs enhanced tamoxifen-induced growth inhibition

To assess the effect of *K pneumoniae* EVs on the ability of tamoxifen to inhibit growth, MCF7 cells were co-treated with tamoxifen and *K pneumoniae* EVs. As expected, tamoxifen alone inhibited cell survival whereas treatment of cells with *K pneumoniae* EVs alone did not affect growth. However, growth inhibition was enhanced when cells were co-treated with tamoxifen and *K pneumoniae* EVs. With tamoxifen alone, cell survival was 47%; however, treatment with tamoxifen and *K pneumoniae* EVs reduced cell survival to 27%. Thus, treatment with *K pneumoniae* EVs resulted in a 43% reduction in cell survival compared to treatment with tamoxifen alone (Fig. 3).

### 3.5 mRNA expression of cyclin E2 was downregulated following co-treatment with tamoxifen and *K pneumoniae* EVs

**Figure 4:**
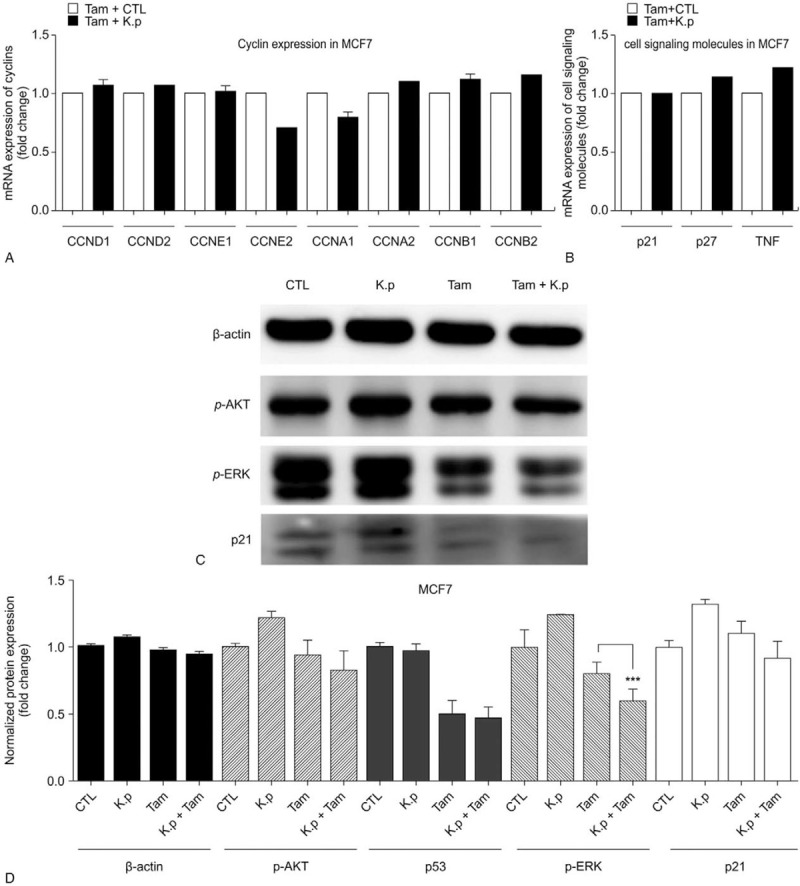
Cyclin E2 and p-ERK mediate *Klebsiella pneumoniea* EV-induced beneficial effect of tamoxifen on MCF7 cells. (A) and (B) Expression of p-AKT1/2/3, p-ERK, p21, and β-actin following treatment with *K pneumoniae* EVs or tamoxifen. MCF7 cells were treated with DMSO (CTL) and 10 μM tamoxifen, 100 ng/ml *K pneumoniae* EVs (*K*.*p*), or 10 μM tamoxifen and 100 ng/ml *K pneumoniae* EVs for 72 h. The expression of p-AKT1/2/3, p-ERK, p21, and β-actin was detected by immunoblotting. Lane 1, control; lane 2, 100 ng/ml *K pneumoniae* EVs; lane 3, 10 μM tamoxifen; and lane 4, 10 μM tamoxifen plus 100 ng/ml *K pneumoniae* EVs. (C) and (D) Expression of cyclin A1, A2, B1, B2, D1, D2, E1, E2, p21, p27, and TNF following treatment with *K pneumoniae* EVs or tamoxifen. Expression of cyclin D1 (CCND1), cyclin D2 (CCND2), cyclin E1 (CCNE1), cyclin E2 (CCNE2), cyclin A1 (CCNA1), cyclin A2 (CCNA2), cyclin B1 (CCNB1), cyclin B2 (CCNB2), p21, p27, and TNF was compared between the tamoxifen and control-treated group (Tam + CTL) and the tamoxifen and *K pneumoniae* EV-treated group (Tam + *K*.*p*). When cells were co-treated with *K pneumoniae* EVs and tamoxifen, expression of cyclin E2 was decreased. ∗∗∗*P* < 0.001. CTL = control, EVs = extracellular vesicles.

mRNA expression analysis of signaling molecules was performed following treatment of cells with tamoxifen and *K pneumoniae* EVs. The expression of cyclins (cyclin D1, D2, E1, E2, A1, A2, B1, and B2), p21, and p27 was examined; mRNA levels of cyclin E2 were decreased following treatment of tamoxifen and *K pneumoniae* EVs. Since *K pneumoniae* EVs are bacterial substances, the inflammatory mediator tumor necrosis factor (TNF) was also studied. The mRNA expression of TNF showed no significant difference in cells treated with tamoxifen only and those co-treated with *K pneumoniae* EVs (Fig. 4A).

### 3.6 Expression of p-ERK and p21 was downregulated by tamoxifen alone as well as by co-treatment with tamoxifen and *K pneumoniae* EVs

To determine the signaling molecules responsible for growth inhibition by tamoxifen or *K pneumoniae* EV plus tamoxifen treatment, we examined the expression of p-AKT1/2/3, p-ERK, and p21. Tamoxifen induced downregulation of p-AKT1/2/3, p-ERK, and p21. When cells were co-treated with *K pneumoniae* EVs and tamoxifen, there was a decrease in p-ERK and p21 compared with tamoxifen alone (Fig. 4B).

## 4 Discussion

The majority of studies of the microbiome have used stool or tissue samples as experimental material. In this study, urine samples were used to investigate microbiome differences. An estimated 99% of the microbial mass in humans is present in the gastrointestinal tract.^[13]^ The microbiome has been directly linked with colon cancer^[14]^ and, thus, feces samples are useful for colon-related diseases.^[15]^ However, the breast is a distinct organ from the colon. Instead of direct interaction with bacteria in the breast, breasts are exposed to bacterial products via bodily fluids. EVs from eukaryotic cells travel throughout the body via bodily fluids, including blood and urine.^[16]^ Bacterial EVs also circulate via body fluids, act on target organs,^[17]^ and ultimately, collect in the urine. Therefore, urine samples were used to investigate the microbiomes in patients with breast cancer.

The microbiomes of breast cancer patients and healthy controls were shown to be statistically different. Moreover, microbiome diversity was greater in the healthy controls than breast cancer patients group. The kinds of microbe in breast cancer patients were reduced, and the proportion of each specific microbe was a large amount compared to that of healthy controls. According to these results, health may be maintained when the composition of the microbiome is balanced to avoid infection or disease. In this study, the breast cancer patients were separated by *Klebsiella* abundance, and the microbiomes of the *Klebsiella*-lacking group exhibited similar diversity to that of the healthy controls. When there is a large amount of a certain microbiome, this result seems to come out because it is close to infection in the human body. In terms of diversity, a small amount of microbiome is diversely present in the healthy control group.

Among microbiomes of breast cancer patients, *Klebsiella* was more abundant in luminal A subtype, estrogen-receptor positive breast cancer patients, and less abundant in healthy controls. Until now, the anticancer effect of plant extracts in breast cancer cell lines has been confirmed by experimental results,^[18]^ or by controlling angiogenesis to promote cancer cell treatment.^[19]^ The anticancer effect of certain ingredients made in bacteria has been identified,^[20]^ but there has never been a paper on the involvement of bacterial EVs in endocrine therapy. When MCF7 cells were treated with *K pneumoniae*-derived EVs alone, cell growth was not significantly suppressed. However, the growth of MCF7 cells co-treated with *K pneumoniae* EVs and tamoxifen was inhibited two-fold compared with tamoxifen treatment alone. These results showed that treatment with *K pneumoniae* EVs enhanced tamoxifen-induced growth inhibition in estrogen receptor-positive breast cancer cells. If *K pneumoniae* is abundant, these bacteria directly affect infection of the human body, as with pneumonia. However, long-term exposure to a small amount of *Klebsiella* EVs, may bring the result in anti-cancer effects.

The estrobolome explains how *Klebsiella* affects hormone therapy of breast cancer. The estrobolome, the collection of enteric bacterial genes, secretes enzymes such as β-glucuronidase and β-galactosidase, which are involved in estrogen deconjugation and reabsorption or secretion from the human body, respectively.^[21]^ The estrobolome can explain how the gut microbiome affects the amount of estrogen and its metabolism in the human body.^[22]^ Bacteria such as *Klebsiella*, *Staphylococcus*, and *Bifidobacterium* secrete these enzymes. *Klebsiella* affects the amount of estrogen in the body by producing β-galactosidase.^[21]^ Therefore, the amount of *Klebsiella* may be related to estrogen levels and the treatment of breast cancer. The abundance of *Klebsiella* observed in breast cancer patient microbiomes is consistent with this theory and the high levels of *Klebsiella* in the luminal A subtype may be linked to the treatment of breast cancer.

The mechanism of interaction between *Klebsiella* and tamoxifen is explained by changes of cyclin in MCF7. Cyclin is associated with the therapeutic efficacy of tamoxifen in hormone receptor-positive breast cancer. According to a previous study, cyclin E2 expression was decreased in MCF7 cells following anti-estrogen therapy, and overexpression of cyclin E2 was related to resistance to anti-estrogen therapy.^[23]^ Therefore, cyclin expression in MCF7 cells was analyzed to elucidate the mechanism of increased tamoxifen efficiency when administered with *K pneumoniae* EVs. This study showed that co-treatment of *K pneumoniae* EVs and tamoxifen downregulated cyclin E2, while cyclin E2 decreased the expression of p21. ERK signaling also appears to be involved. In a previous study, cyclin E-CDK2 binding was shown to play a role in the phosphorylation of cyclin-dependent kinase inhibitors, such as p21 or p27.^[24]^ These pathways are consistent with the data obtained from cells co-treated with *K pneumoniae* EVs. p21 plays a role in carcinogenesis by inhibiting apoptosis in breast cancer.^[25]^ It is possible that *K pneumoniae* EVs intensified the tamoxifen-induced downregulation of cyclin E2 and inhibited the growth of MCF7 cells by decreasing the production of p21 protein. According to Ciccarelli et al, the expression of p21 is driven by the MERK/ERK pathway.^[26]^ ERK signaling is known to promote cell survival by increasing the expression of pro-survival proteins.^[27]^ Increased ERK signaling has been linked to tamoxifen resistance, and combination therapy using tamoxifen and regulation of ERK signaling has been shown to be effective in tamoxifen-resistant cells.^[28]^ Downregulation of p-ERK may be related to the reduced survival of MCF7 cells and may increase tamoxifen efficacy.

The microbiome of *Klebsiella* EV enhanced the effect of tamoxifen. The limitation of this study is that we do not know which metabolites of EV increase tamoxifen efficacy. Further studies are necessary using multiomics approaches to determine the target metabolites in the *Klebsiella* EVs. Another limitation is that one cell line is not sufficient to show that *Klebsiella* EVs influence tamoxifen treatment. A more detailed understanding of the observed effects of the EVs and in vivo confirmation will be required. However, to the best of our knowledge, this is the first demonstration of the ability of bacterial EVs to increase tamoxifen efficacy.

In conclusion, this study shows that microbiome-derived *K pneumoniae*, an important bacteria in breast cancer patients, has an impact on tamoxifen-induced effects and the expression of some signaling molecules in cultured MCF7 cells. This study suggests the possibility that changes in the microbiome may be useful as an alternative pharmabiotic strategy in the future.

## Author contributions

**Conceptualization:** Jeongshin An.

**Data curation:** Nam Sun Paik Paik, Woosung Lim, Byung-In Moon.

**Formal analysis:** Jeongshin An.

**Investigation:** Jeongshin An, Jong Bin Kim, Eun Yeol Yang.

**Methodology:** Jeongshin An, Jinho Yang, Won-Hee Lee, Yoon-Keun Kim.

**Project administration:** Jeongshin An.

**Resources:** Jeongshin An, Jinho Yang, Won-Hee Lee, Yoon-Keun Kim.

**Supervision:** Byung-In Moon.

**Writing – original draft:** Jeongshin An.

**Writing – review & editing:** Jeongshin An, Hye Ok Kim, Hyungju Kwon.
